# Reuben1If the “gentle” reader will but turn to his Bible, if he hath one, and read the first three words of the fourth verse of the forty-ninth chapter of Genesis, he will readily observe how appropriately the eldest son of Jacob stands as the captain of these “few lines.” He will please read no further, lest what follows those three words may suggest what is uncomplimentary to the writer.

**Published:** 1878-04

**Authors:** Ausburn Towner


### REUBEN.[1]

[WITH COPIOUS NOTES AND SOMEWHAT AFTER THE MANNER OF THE HEAVY QUARTERLIES.]

BY AUSBURN TOWNER.

The thoughtful man, when observing the operations of mind or matter, whether in their relations to cycles of time or to one dot of a generation, must be struck with awe at the utter instability of them all. There is a constant shifting,[2] an appalling absence of rest and quiet, that the more steadily we regard them, seems the more terrible. The mere idea of incessant and eternal motion and change, is one that we cannot entertain with any more satisfaction than we can the ideas, if we call them so, of eternity or infinite space. Try it and see how soon the brain will begin to whirl, or the senses, finite as they are, will take refuge in sleep.[3] The voluntary actions of our bodies may cease and we may think we lie down and rest, but the involuntary actions that continue our lives never cease until the ever-changing tissues 'wear out,' as it is called, and we rest indeed.[4] We are pointed to the stars and told that these, at least, remain stable, unaffected by time; that 'belted Orion' was there and that the 'sweet influences of the Pleiades' are no more perceptible to us than they were to Job, who lived so many years ago that his time is lost

1. If the "gentle" reader will but turn to his Bible, if he hath one, and read the first three words of the fourth verse of the forty-ninth chapter of Genesis, he will readily observe how appropriately the eldest son of Jacob stands as the captain of these "few lines." He will please read no further, lest what follows those three words may suggest what is uncomplimentary to the writer.

2. The Yankees are Nature's choicest examples of her own restless activity. They are her pet children—always shifting and moving. The most bitter word in the whole New England dialect is "shiftless," meaning unchangeable or stationary, and not, as 'Aunt Ophelia' put it in 'Uncle Tom's Cabin' in referring to the unclothed condition of Topsy.

3. One method prescribed by physicians for producing sleep in a healthy organization, is for the sleep-wisher to shut his eyes and imagine that he sees an immense wheel making an endless number of revolutions. Sleep must come or the wheel must stop.

4. A business man of upwards of seventy years of age, who had lived a life of incessant activity, being applied to by his friends to take some rest from his labors, replied very aptly, "Rest? Rest, my dear sir, I shall have an eternity to rest in."

among the deep shadows of antiquity. Indeed! Let us see for ourselves. Even so late as the discovery of the magnetic needle with us, for the far Eastern nations knew its properties so long ago that we cannot compute the period of its earliest use among them, it pointed directly towards the north star. Does it do so now? Try it and see. There is a perceptible deviation even when it is not acted upon by other influences. Give it time and it will point the other way. It *could not* continue to do precisely the same thing forever. It is subject to the same instability that marks all things. Then there is a method to find this same star, which has come down to us from the Chaldeans and Magians of ages ago, as well known now in our nurseries as to the schoolmen. There are two large stars in the constellation of 'Ursa Major' or 'The Great Bear,' which have been called 'The Pointers' since they show where 'Polaris' shines. A straight line drawn through them, we have been taught, and continued, will reach that 'mariners' safety lamp.' It might have done so once. It would make one cross-eyed almost, now to think it true. You will have to make the line take quite a considerable curve to take in the north star these days.

So far as material things are concerned their instability is a matter that needs no argument, though it is as susceptible of as many illustrations as there are infinitesimal atoms in the universe. But the equal instability of ideas, or as they are called, 'emanations from the brain,' if not more generally known, are more curious and astounding.

Some philosophers contend that ideas are not evolved from an inner consciousness called the brain, but are produced by the action of matter on that organ, so that there could never be such a thing, abstractly, as an invention, everything being merely a discovery; making us all objective, not subjective beings, originating in ourselves nothing, experiencing all we know.[5] If this were so, the changeableness or instability of matter, would, of course, be reflected from all things upon which it acted, and its properties would also be thereby transferred. Believing, however, that ideas exist independently of matter, their instability becomes seriously surprising. What changes them? For a thought would seem to be immortal.

As, for instance, the idea that early rising is conducive of health—a notion that is embodied in the ancient quatrain 'Early to bed,' &c.—once obtained universal acquiescence. Now we hear of unwholesome miasmatic influences that creep close to the earth during the night time, and are not lifted away from the reach of the respiratory organs until the beneficent sun has attracted them into the regions of the clouds. Was this invented or discovered for the sake of some pecunious invalid who loved a good long morning nap and a nine o'clock breakfast as soon as he arose, or is it only of late that our world has developed a night sweat that breeds disease? I know of one individual who had a very flexible elbow, who, in my youth put little faith in such a notion.[6]

Again, the idea was once prevalent that in the matter of eating it were well to be governed by good sense, some reputable physicians teaching that it was even better to get up from the table hungry than to eat all your appetite demanded. A later view of the subject, enunciates the principle that it is best to eat all that you can hold, even doing as the little boy did, standing up to get more down, than to give it up before you are full. Is this also for the benefit of some pecunious one, who wanted a physician's certificate to excuse himself for being a glutton?

Still again, disease has heretofore been considered as an involuntary sort of a thing, coming like love. You could catch it, perhaps, or inherit it, but you could not, by any act of your own, pro-

---

5. Schlegel's Matter and Mind, p. 461.

6. These old-fashioned notions of our fathers, in another channel of thought, are more amusing than this one or those that follow. For example: I have heard men, who by no means think the sum of their lives has been added up, who have an idea that with the passing away of their generation, will pass away all that the world knows of excellence. You should hear them talk of the actors and actresses upon whom the foot-lights shone in their day; of a certain scrawny, bony, tired old woman, for she never came to this country until her charms and her reputation were on the wane, to whom they refer as the 'divine Fanny,' but who appeared on the bill boards, and in the advertisements simply as 'Fanny Ellsler.' "Surely, old boy, she was a paragon, and the world will never look upon her like again." It is amusing to hear them bewail the degeneracy of these days, and see how their eyes light up when you mention to them the Old Park Theatre of New York, which was burned before many of us were born, and which, at its best, was as inferior in all of its appointments to the places of public amusement of these days, as were the barn entertainments of the days of Charles I., of England, to it. The truth is, this instability, this perpetual action and effort suggests something better and compels improvement. It could not be otherwise. Every day sees that which is as much better than the day it succeeds, as railroad traveling is better than bumping along in the old stage-coach, notwithstanding the fact that similar old fellows to the ones alluded to above, *will* continue to bore the patient listener about those 'good old times.' There never was any one born, so absolutely necessary to the world, that when he died some stronger and better man did not take his place. When Dean Richmond died, and he is almost forgotten now, there was a positive dread along the line of the New York Central Railroad that that corporation would not hold together; that there was no one in the wide world, who could fill his place at the head of its management. Yet what was Dean Richmond in comparison to Vanderbilt? Joseph Addison may have been a good essayist for his time. Can you say so from having read him, my friend? But in comparison with George William Curtis of our day and generation, he is but saw-dust and chips beside roast beef and plum pudding. Set him down to the work that Curtis has done so admirably for years, and you'd speedily find him 'running to emptin's.'

duce it, or create it. Now, at length, one sad complaint has been invented or discovered, which a man can make: a voluntary disease, dependent upon the will.[7] It is called Alcoholism. Is this also for some pecunious one who wanted a scientific flag to hold over himself when he got drunk? Is disease a matter of the will? How comes it that the exercise of the will will cure it, then, as, thank God! it has done in so many instances in this neighborhood lately? Why don't the doctors go to work and cure consumption or scrofula or the measles or other diseases by the same course of treatment?[8] The appetite, the insatiable craving for strong drink is bad enough when created, in all conscience, without miscalling it, and dignifying an evil and a sin with a polite but incorrect term, making it seem to be what it is not, a disease in either the usual or professional acceptation of the term.

Once more: An idea has very extensively prevailed, nurtured, of course, by those most interested in its propagation and continuance, that we owe, very largely, the revival of learning in the Middle Ages, to the monks; that in their monastaries were preserved through what are known as the "Dark Ages" all the enlightenment and knowledge that prevailed before the Huns and Goths sat down so hard upon the Roman world. This is *certainly* an idea, a figment of the brain, something evolved from the inner consciousness, with neither materialism nor truth about it. All that we have which is really of practical use, came to us from the Arabs, through the Moors. The ancient world had no more useful schools or schools of more practical value, than the famous one at Cordova or the others equally well known in Granada. The Romans and Greeks only speculated; they never experimented nor proved. While they, with their imaginations, were filling the heavens with the spirits of their heroes and their Gods, the Arabians were calculating eclipses, watching the passage of Venus over the disc of the sun, naming the stars, and measuring their distances from us. The Arabian physicians, astronomers and scientific men generally, were as much superior to those of Rome and Greece as the Court of Caliph Haroun Alraschid excelled in cultivation and splendor the Court of his contemporary Charlemagne of France. In such brief space as the BISTOURY allows for rumination, it would be impossible to specify or illustrate at large, but take just one specimen brick. In mathematics, for instance, how much of a calculation could be made with the Roman numerals? Multiply XXIV by XIX. How would you or Euclid go to work at that job? The Arabic figures 1, 2, 3, 4, 5, 6, &c., were a power that those who dwelt on the banks of the Tiber or on the shores of the Ægean sea never possessed. The Arabs were at least *that* much ahead of them, and not alone in that by any means.[9]

I have said that the contemplation of the instability, the constant changeableness of all things, is sufficient to fill the mind of the thoughtful man with awe and wonder. In a much greater degree will the opposite of this produce a like effect. The idea of eternal peace and quiet, no bustle, no fuss, no hurry, no change, enters largely into our most elevated aspirations, our notions of Heaven and God. The place must be totally different from this, that we have here; the person altogether unlike man, and we seem to draw nearer to the one and feel more strongly the presence of the other when we approach that which is the furthest removed from noise or motion. Stand upon one of our western prairies on a still summer day, out of sight of everything save the green earth and the blue sky; go to some dense forest, where even the lightest breath of air is not stirring and where the stillness is almost painful; or rest for a moment in a desert where the hot sand reaches from your feet in all directions until it meets the burning sky at the horizon, and a sentiment will fill your breast, you cannot help it, that, somehow, even if you do not understand it, as you are farther away from man and the things of the world, so are you nearer to God and His Dwelling Place.

---

7, A man was eating dumplings with maple sugar. He had already made away with some twenty or more of the delicious viands, and his little son had repeatedly passed up his plate to assist at the repast, only to be disappointed at each application. "You can't have any, my son," said the appreciative mother. "Papa's sick!" What was his disease?

8. "Do you call yourself a doctor?" said an offended man to a learned physician recently. "I do," was the modest response. "And do you practice?" continued the questioner. The same answer followed. "You must have an immense success," pursued the irate man. "Why?" replied the flattered disciple of Galen and Esculapius. "Oh, you're so almighty mean," concluded the angered one, "that any decent disease would dig out if you should come within gun-shot of it.

9. In this connection, I cannot help remarking, what seems to be but little, if at all known, that the invention or discovery of the use of the cipher or naught (0) is of comparatively recent date, being not yet five hundred years old. It was one of the most important of events, giving the world one more figure, the most powerful of all of them, and advancing it by one step, leagues on the way to decimal notation. Before this use of the cipher (0) the power of the figures was determined by perpendicular lines, thus:—

| |5| |4| |

Which reads 5040, or five thousand and forty. Thoughtless ones are to be found who sneer at the idea of the power of Infinite wisdom to create something out of nothing, yet here finite man has produced untold strength and force from 0.

